# Microbiomes of Velloziaceae from phosphorus-impoverished soils of the *campos rupestres*, a biodiversity hotspot

**DOI:** 10.1038/s41597-019-0141-3

**Published:** 2019-07-31

**Authors:** Antonio Pedro Camargo, Rafael Soares Correa de Souza, Patrícia de Britto Costa, Isabel Rodrigues Gerhardt, Ricardo Augusto Dante, Grazielle Sales Teodoro, Anna Abrahão, Hans Lambers, Marcelo Falsarella Carazzolle, Marcel Huntemann, Alicia Clum, Brian Foster, Bryce Foster, Simon Roux, Krishnaveni Palaniappan, Neha Varghese, Supratim Mukherjee, T. B. K. Reddy, Chris Daum, Alex Copeland, I.-Min A. Chen, Natalia N. Ivanova, Nikos C. Kyrpides, Christa Pennacchio, Emiley A. Eloe-Fadrosh, Paulo Arruda, Rafael Silva Oliveira

**Affiliations:** 1 Centro de Biologia Molecular e Engenharia Genética, Universidade Estadual de Campinas (UNICAMP), 13083-875 Campinas, SP Brazil; 2 Departamento de Genética e Evolução, Instituto de Biologia, Universidade Estadual de Campinas (UNICAMP), 13083-875 Campinas, SP Brazil; 3 Genomics for Climate Change Research Center, Universidade Estadual de Campinas (UNICAMP), 13083-875 Campinas, SP Brazil; 4 Departamento de Biologia Vegetal, Instituto de Biologia, Universidade Estadual de Campinas (UNICAMP), 13083-862 Campinas, SP Brazil; 5 Embrapa Informática Agropecuária, 13083-886 Campinas, SP Brazil; 6 Instituto de Ciências Biológicas, Universidade Federal do Para (UFPA), 66075-750 Belem, PA Brazil; 7 School of Biological Sciences, University of Western Australia (UWA), Perth, WA 6009 Australia; 8 Department of Energy Joint Genome Institute, Walnut Creek, California, 94598 USA

**Keywords:** Microbiome, Plant symbiosis

## Abstract

The rocky, seasonally-dry and nutrient-impoverished soils of the Brazilian *campos rupestre*s impose severe growth-limiting conditions on plants. Species of a dominant plant family, Velloziaceae, are highly specialized to low-nutrient conditions and seasonal water availability of this environment, where phosphorus (P) is the key limiting nutrient. Despite plant-microbe associations playing critical roles in stressful ecosystems, the contribution of these interactions in the *campos rupestres* remains poorly studied. Here we present the first microbiome data of Velloziaceae spp. thriving in contrasting substrates of *campos rupestres*. We assessed the microbiomes of *Vellozia epidendroides*, which occupies shallow patches of soil, and *Barbacenia macrantha*, growing on exposed rocks. The prokaryotic and fungal profiles were assessed by rRNA barcode sequencing of epiphytic and endophytic compartments of roots, stems, leaves and surrounding soil/rocks. We also generated root and substrate (rock/soil)-associated metagenomes of each plant species. We foresee that these data will contribute to decipher how the microbiome contributes to plant functioning in the *campos rupestres*, and to unravel new strategies for improved crop productivity in stressful environments.

## Abstract (summary)

**Table:** 

Design Type(s)	strain comparison design • stimulus or stress design
Measurement Type(s)	soil metagenome
Technology Type(s)	DNA sequencing • amplicon sequencing
Factor Type(s)	
Sample Characteristic(s)	plant metagenome • Brazil • soil • Vellozia epidendroides • leaf • root • rhizosphere • stem • rock • Barbacenia

Machine-accessible metadata file describing the reported data (ISA-Tab format)

## Background & Summary

**Fig. 1:**
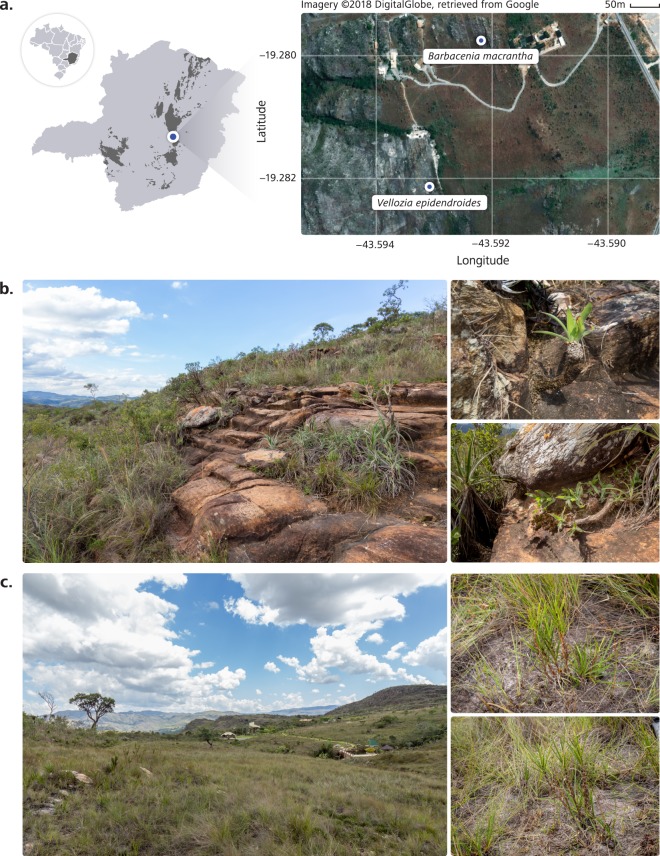
The Brazilian *campos rupestres* are rocky seasonally-dry environments with some of the world’s most phosphorus (P)-impoverished soils. (**a**) The study was conducted in a *campo rupestre* site in the Brazilian state of Minas Gerais, as shown on the map (left). *Campo rupestre* areas are shown in dark gray. The sites where plants of each Velloziaceae species were collected are indicated in the aerial image of the study area (right). (**b**) *Barbacenia macrantha* was found in a rocky area (left), where it grows over exposed rocks (right). (**c**) *Vellozia epidendroides* specimens were collected in an area (left) where they grow in patches of shallow soil (left).

The Brazilian *campos rupestres* are an ecoregion located on the rocky outcrops of central and eastern regions of Brazil (Fig. 1a)^1^. Most *campos rupestres* occur along the Espinhaço range, a Proterozoic Quartzite formation, with slow-disintegrating parent material^2^. Despite containing some of the world’s most P-impoverished soils^3^, the *campos rupestres* are a biodiversity hotspot that harbors exceptional diversity and endemism. Even though they occupy less than 1% of the Brazilian land area, the *campos rupestres* host more than five thousand vascular plant species, over 40% of which are endemic to this ecosystem^4^. Although several ecophysiological studies on plant species of *campos rupestres* have been conducted, the contribution of microbial communities to plant survival in such stressful conditions remains elusive.

A dominant monocot plant family, Velloziaceae, displays remarkable success in this environment. Members of this group display strategies to cope with extremely nutrient-poor soils, such as efficient P remobilization from senescent leaves, the formation of rhizosheaths and vellozioid roots, which exhibit root-mediated carboxylate secretion that enhances nutrient uptake^5,6^. These adaptations allow Velloziaceae to grow on P-impoverished substrates with different properties, such as exposed rocks (Fig. 1b) and shallow patches of soil (Fig. 1c).

Association with mycorrhizal fungi is one of the most ancient, widespread and important symbiosis for uptake of P and other nutrients, being found in over 80% of vascular species^7^. However, it has been suggested that in severely P-impoverished soils, such as those in the *campos rupestres*, the costs of maintaining mycorrhizal associations exceed their nutrient uptake benefits^8^. Consequently, most plants growing on the *campos rupestres* do not exhibit mycorrhizal association^3,5^. While the absence of mycorrhizal fungi raises the question as to whether other microbial associations are beneficial, to our knowledge no previous study has investigated the extent of phylogenetic and functional diversity of microbial communities in the *campos rupestres*. As a result, the functional role of the microbial communities associated with native species thriving in different *campos rupestres* environments remains obscure.

**Fig. 2:**
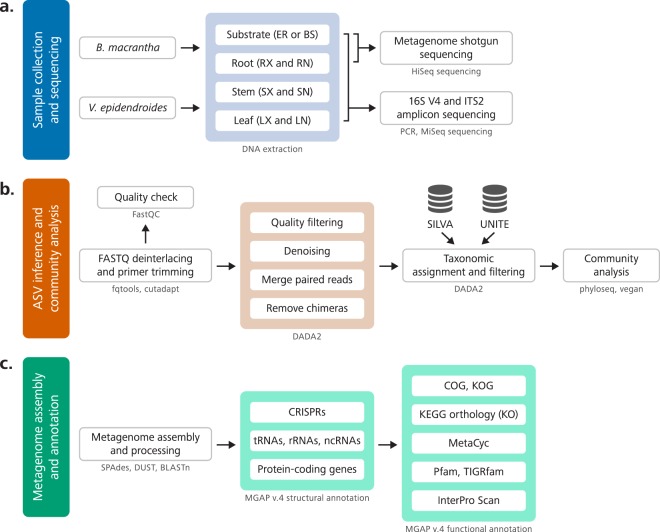
Overview of the workflows used to obtain and process the data. (**a**) Six individuals of both *Vellozia epidendroides* and *Barbacenia macrantha* were collected from their natural habitats and individually processed to assess the microbiomes from seven different environments through extraction of microbial DNA. The DNA extracted from three samples of four distinct communities (*B*. *macrantha* substrate, *B*. *macrantha* rhizosphere, *V*. *epidendroides* substrate and *V*. *epidendroides* rhizosphere), totaling 12 samples, was sequenced on an Illumina HiSeq platform to generate data for the metagenomic assembly. DNA from all six samples of all the assessed communities, totaling 84 samples, was used to generate 16S V4 and ITS2 amplicons, which were sequenced on an Illumina MiSeq platform. BS = bulk soil, ER = exposed rock, RX = rhizosphere, RN = endophytic root, SX = exophytic stem, SN = endophytic stem, LX = epiphytic leaf, LN = endophytic leaf. (**b**) The microbial community analysis started with the removal of primer sequences from the sequenced amplicons. Next, reads were denoised using the DADA2 pipeline, and the identified ASVs were assigned to bacterial and fungal taxa though comparison with the SILVA and UNITE databases, respectively. After filtering out ASVs from mitochondria and chloroplasts and low-prevalence amplicons, the phyloseq and vegan packages were used to analyze community composition. (**c**) The metagenomes were assembled using SPAdes software and then annotated using the standard DOE-JGI MGAP v.4 annotation pipeline. In the structural annotation step, the metagenomes were surveyed to identify CRISPRs, tRNA genes, rRNA genes, other classes of ncRNA genes and protein-coding genes. Next, the protein-coding sequences were functionally annotated and assigned to ortholog groups, metabolic pathways, chemical reactions and protein families.

Aiming to uncover the composition and functional role of Velloziaceae-associated microbial communities, we surveyed the microbiota associated with two Velloziaceae species that thrive in two nutrient-impoverished (Tables 1 and S1) substrates: *Barbacenia macrantha* Lem and *Vellozia epidendroides* Mart. ex Schult. & Schult. f., growing on rocks (Fig. 1b) and in soil patches (Fig. 1c), respectively. Rock and soil substrates surrounding the individuals, and epiphytic and endophytic compartments of their roots, stems and leaves (Fig. 2a) were sampled for profiling the microbial community through sequencing of the 16S V4 rRNA region, for prokaryotes, and ITS2, for fungi (Fig. 2b). We assessed the genic landscape through metagenome sequencing of substrate and rhizosphere communities (Fig. 2c).

**Table 1:** Physicochemical characterization of soil and rock samples from the study sites of *Vellozia epidendroides* and *Barbacenia macrantha*.

Substrate	pH	Organic matter (g/kg)	N (mg/kg)	P (mg/kg)	K (mg/kg)	Ca (mg/kg)	Mg (mg/kg)	S (mg/kg)
Soil	3.55 (0.06)	39.90 (5.92)	900.00 (270.80)	4.15 (2.52)	34.32 (9.07)	129.00 (17.81)	14.18 (0.51)	4.10 (2.25)
Rock	4.74 (0.11)	6.67 (0.11)	60.00 (54.77)	1.21 (0.60)	47.19 (20.41)	66.53 (8.55)	8.08 (0.27)	2.36 (2.02)

pH and concentrations of organic matter and macronutrients (N, P, K, Ca, Mg, and S) of soil and rock are shown. Values correspond to the means of five samples. Standard deviations are in parenthesis.

**Fig. 3:**
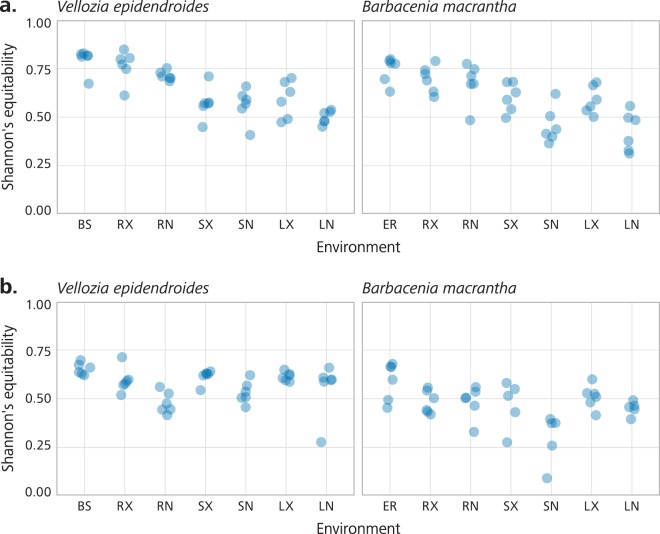
Alpha diversity of the *Vellozia epidendroides* and *Barbacenia macrantha* microbiomes. Alpha diversity, quantified using Shannon’s equitability index, of the (**a**) 16S V4 and (**b**) ITS2 loci retrieved from several microbial communities associated with *V*. *epidendroides* and *B*. *macrantha*. BS = bulk soil, ER = exposed rock, RX = rhizosphere, RN = endophytic root, SX = exophytic stem, SN = endophytic stem, LX = epiphytic leaf, LN = endophytic leaf.

**Fig. 4:**
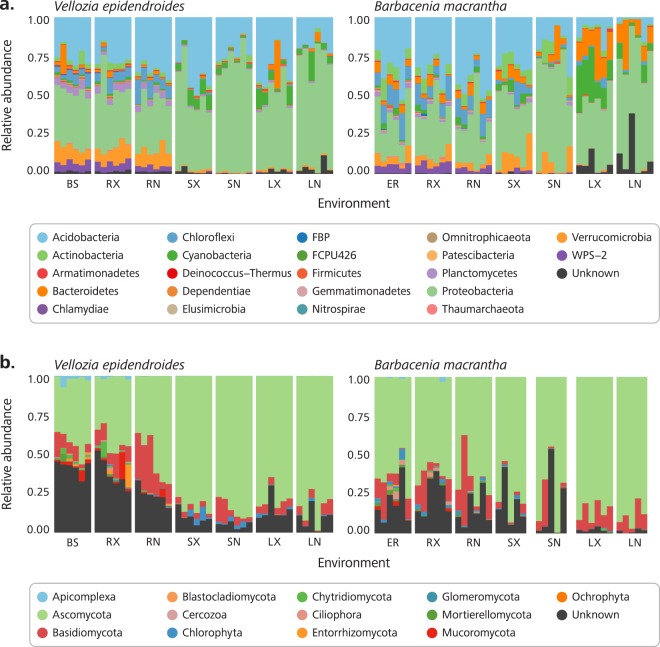
Community composition of the *Vellozia epidendroides* and *Barbacenia macrantha* microbiomes at the phylum level. Relative abundance of (**a**) prokaryotic and (**b**) fungal phyla retrieved from 16S V4 and ITS2 amplicon sequencing, respectively. Each column represents a single sample and samples were grouped according to the environment from which the communities were accessed. BS = bulk soil, ER = exposed rock, RX = rhizosphere, RN = endophytic root, SX = exophytic stem, SN = endophytic stem, LX = epiphytic leaf, LN = endophytic leaf.

High-throughput sequencing of DNA fragments amplified from the 16S V4 and ITS2 regions was produced for 84 16S V4 and 81 ITS2 samples^9^, with median read number of 123,496 and 199,968, respectively (Supplementary Table S2). Processing of these data retrieved 28,582 and 10,981 amplicon sequence variants (ASVs) from the 16S V4 and ITS2 regions, respectively. Analysis of the ASV abundance revealed that, for both bacteria and fungi, community diversity was tied to the environment (Fig. 3). The prokaryotic diversity (Fig. 3a) was generally higher than the fungal diversity (Fig. 3b). We also found that most of the 16S V4 amplicons were assigned to at least one of the 22 identified prokaryotic phyla (Fig. 4a), while a substantial fraction of the ITS2 sequences could not be classified. In the case of ITS2, a single phylum, among the 13 identified phyla, encompassed most of the sequenced amplicons (Fig. 4b).

Shotgun sequencing of total DNA extracted from microbial samples of rhizosphere and substrate generated a total of 192 GB of sequencing data. The samples were individually assembled, producing 12 metagenomes with a median assembly length of 918,800,525 bp, a median scaffold number of 2,121,680 bp and a median N50 of 536,506 bp (Table 2). Annotation of those metagenomes retrieved a median number of 9,907 noncoding genes and 2,544,611.5 protein-coding genes. The comparison between metabolic profiles of communities associated with the substrates and the rhizospheres of the two plants revealed major differences between the two environments (Supplementary Fig. S1). We found that 271 and 104, out of 1,403, MetaCyc pathways are differentially abundant (FDR < 0.05) between soil and rock-associated and between *V*. *epidendroides* and *B*. *macrantha*-associated communities, respectively (Supplementary Fig. S2).

**Table 2:** Metagenome assembly and annotation statistics.

	Vellozia epidendroides						Barbacenia macrantha					
	Bulk Soil			Rhizosphere			Exposed Rock			Rhizosphere		
	BS_R01	BS_R02	BS_R03	RX_R1	RX_R2	RX_R3	ER_R07	ER_R08	ER_R09	RX_R7	RX_R8	RX_R9
**Assembly length (bp)**	860,879,893	2,268,702	617,499,457	676,518,752	976,721,157	729,110,140	600,610,973	1,214,420,372	1,238,859,002	1,079,199,799	1,433,396,097	1,622,069,667
**Number of contigs**	1,972,903	2,270,457	1,351,797	1,486,891	1,645,436	1,645,662	1,377,103	2,326,200	2,637,801	2,492,579	2,699,276	2,952,973
**N50**	614,578	598,284	407,288	452,936	317,068	503,998	432,757	569,015	717,185	61,759	628,284	673,204
**L50**	406	490	432	287	645	416	408	516	441	405	520	551
**Max scaffold length (bp)**	59,246	1,680,496	258,893	27,505	662,532	1,657,979	327,896	651,618	2,357,837	61,759	2,793,540	1,186,326
Genes												
RNA genes	8,700	10,837	6,182	6,561	9,412	8,158	7,331	11,198	13,860	10,402	13,115	14,833
rRNA genes	2,332	2,630	1,866	1,809	1,815	2,368	2,019	2,360	3,399	2,706	2,488	3,100
5S rRNA	146	252	133	137	252	172	151	249	294	212	264	310
16S rRNA	711	786	533	545	540	699	657	672	1,027	798	707	945
18S rRNA	32	40	54	51	54	42	22	84	68	67	84	69
23S rRNA	1,366	1,477	1,031	987	870	1,394	1,155	1,214	1,891	1,514	1,300	1,665
28S rRNA	77	75	115	89	99	61	34	141	119	115	133	111
tRNA genes	6,368	8,207	4,316	4,752	7,597	5,790	5,312	8,838	10,461	7,696	10,627	11,733
Protein coding genes	2,297,228	2,791,995	1,590,322	1,754,025	2,150,110	1,926,163	1,628,300	2,880,790	3,166,526	2,901,223	3,366,379	3,764,853
with Product Name	2,305,928	2,802,832	1,596,504	1,760,586	2,159,522	1,934,321	1,635,631	2,891,988	3,180,386	2,911,625	3,379,494	3,779,686
with COG	1,191,680	1,496,157	825,877	942,870	1,186,733	1,014,644	874,346	1,446,673	1,606,760	1,515,597	1,722,337	2,006,246
with Pfam	1,109,275	1,412,963	768,892	875,344	1,126,410	943,934	805,481	1,396,975	1,521,778	1,418,186	1,659,192	1,927,535
with KO	913,196	1,137,348	621,315	720,502	888,464	773,464	675,421	1,076,987	1,224,775	1,174,302	1,300,850	1,519,429
with Enzyme	550,718	672,962	377,026	431,594	511,776	472,682	410,088	657,136	753,442	715,698	788,012	917,051
with MetaCyc	351,479	427,878	241,918	276,579	321,172	304,400	261,797	422,831	483,869	459,140	508,562	585,951
with KEGG	569,005	700,632	386,214	449,908	539,886	484,161	423,557	663,022	763,289	732,531	804,817	940,101
**COG clusters**	4,106	4,227	4,002	4,075	4,182	4,133	4,115	4,281	4,344	4,240	4,327	4,375
**Pfam clusters**	6,383	7,003	5,946	6,250	6,890	6,355	6,261	7,400	7,263	7,030	7,870	7,445
**CRISPR count**	375	350	233	272	280	308	220	741	666	502	904	755

BS = bulk soil, ER = exposed rock.

These data are the result of the first effort to explore microbiomes of the *campos rupestre*s and have the potential to uncover novel functional roles of plant-associated microbial community. We expected it to be relevant to both the understanding of the role of microorganisms in plant survival and the development of novel strategies to improve crop productivity in stressful environments.

## Methods

### Study site characteristics and plant species

Plant samples were collected on March, 2017 in “Reserva Natural Particular Vellozia” (19°16′55.8″S 43°35′34.9″W and 19°16′47.1″S 43°35′32.0″W for *V. epidendroides* and *B. macrantha*, respectively; Fig. 1a), a private natural reserve adjacent to the Serra do Cipó National Park, Minas Gerais, Brazil. This site is located in the *Espinhaço* range, a rupestrian habitat characterized by rock outcrops and sandy soils with low availability of nutrients, especially P^4^, which was ascertained by physicochemical characterization of rock and soil samples (Tables 1 and S1). This site was chosen because of the occurrence of Velloziaceae species in two distinct microhabitats, *B*. *macrantha* growing on exposed rocks (Fig. 1b) and *V*. *epidendroides* growing in patches of shallow soil (Fig. 1c).

### Sample collection

To assess the composition and structure of microbial communities associated with epiphytic and endophytic compartments of *V*. *epidendroides* and *B*. *macrantha*, we sampled roots, stems, leaves and surrounding soil/rocks from six individuals of each plant species in March of 2017 in a total of 84 samples (Supplementary Tables S3 and S4). For each environment, we defined an area of approximately 200 m^2^ within which we collected plant and soil/rock materials. To make sure that we would sample plants that were representative of these environments, we defined the boundaries so that the areas were as visually consistent as possible. During the sampling process, the chosen specimens of each plant species were randomly assigned sample numbers from R1 to R6.

*V*. *epidendroides* plants were sampled from a large population in the shallow soil area (Fig. 1a,c). Individuals similar in height, number of leaves and number of tillers were chosen. Each plant was excavated from the soil by inserting an ethanol-sterilized shovel to a depth of 15 cm in a circular perimeter with a 20 cm radius around the plant. The entire plant was lifted and placed in a sterile, labeled container. The leaves were hand detached from the stem, stored in plastic bags and placed on ice for further processing. The stem was separated from the roots using ethanol-sterilized pruning scissors and kept inside plastic bags on ice. Roots were manually shaken to remove large soil aggregates and stored in the same manner.

*B*. *macrantha* plants were sampled in a rocky slope area (Fig. 1a,b) and individuals were chosen based on the same criteria used for *V*. *epidendroides*. Plants were removed by breaking the surrounding rock with an ethanol-sterilized hammer and chisel until roots were exposed. Pieces of rocks were collected in plastic bags and placed on ice. Before microbiome sampling, rocks were crushed to small pieces. Plants harvested from rocks had their leaves, stems and roots sampled and stored on ice using the same procedures described for *V*. *epidendroides*.

Microbes were collected from plant organ samples by methods adapted from a previously described protocol^10^. Briefly, the epiphytic microbial community was obtained by washing the root, stem and leaf samples in sterile ice-cold 1× PBS (7 mM Na_2_HPO_4_, 3 mM NaH_2_PO_4_ and pH 7.0) with 0.05% (v/v) Tween 20 buffer solution. The same washing procedure was applied to grinded pieces of rocks to assess microbial communities of exposed rocks. Soil samples were directly submitted to DNA extraction without further processing. The washing solution was centrifuged at 3,000 × g for 15 min at 4 °C, and the resulting pellet, defined as the sample containing enriched epiphytic microbial communities, was frozen in liquid nitrogen and stored at −80 °C. The washed plant organs were subjected to a second washing step to remove the remaining buffer solution. Plant organs were cut and blended in ice-cold 1× PBS buffer solution. The blended buffer was centrifuged at 200 g for 5 min at 4 °C to remove particulates and cell debris. The supernatant was then centrifuged at 3,000 × g for 15 min at 4 °C. The resulting pellet, defined as the sample containing an enriched endophytic microbial community, was frozen in liquid nitrogen and stored at −80 °C.

We also sampled soil and rock material for physicochemical characterization. The samples were obtained in the original study areas in June, 2018. Extraction of the material was done within 20 cm of a *V*. *epidendroides* or *B*. *macrantha* individual, following the same sampling procedures used for microbiome assessment.

### Physicochemical characterization of soil and rock samples

To prepare samples for physicochemical characterization, rocks were first ground to fine particles. Next, pulverized rock and soil samples were individually air-dried and sieved (<2 mm) to remove large particles and organic remains. The nutrient content and physical properties of the processed material were determined at the Agronomy Institute (IAC), in Campinas, following standardized methods^11^.

Briefly, phosphorus (P), calcium (Ca), magnesium (Mg), and potassium (K) were extracted using ion exchange resins^12^ and quantified by colorimetry (P), atomic absorption spectrophotometry (Ca and Mg), and flame photometry (K). Aluminium (Al) was extracted with potassium chloride solution and quantified using titration. Sodium (Na) was extracted with ammonium acetate solution (pH 7.0) and measured by flame photometry. Boron (B) was extracted with hot water and determined through spectrophotometry. Copper (Cu), iron (Fe), manganese (Mn), zinc (Zn), cadmium (Cd), lead (Pb), chromium (Cr), and nickel (Ni) were extracted using the diethylene triamine pentaacetic acid method^13^ and quantified with inductively coupled plasma optical emission spectrophotometry. Total nitrogen (N) was extracted and quantified using Kjeldahl method^14^. Organic matter content was determined through dichromate oxidation followed by colorimetry^15^. The pH was quantified in CaCl_2_-diluted (0.01 M) samples. SMP-pH and exchangeable acidity were determined by dilution of the samples in SMP buffer solution^16^. To quantify the electrical conductivity, 100 g of soil was ressuspended in 100 mL of deionized water and the resulting solution conductivity was measured with an electrical conductivity meter.

### DNA extraction, amplicon and shotgun metagenomic sequencing

DNA was extracted from enriched microbial samples using a PowerSoil DNA Isolation kit (MO BIO Laboratories, Inc., Carlsbad, CA, USA) with minor modifications to the default protocol as previously described^10^. Extracted DNA quality was assessed by a NanoDrop spectrophotometer (Thermo Fisher Scientific Inc., MA, USA) and quantified by a Qubit dsDNA BR Assay Kit (Thermo Fisher Scientific Inc., MA, USA) prior to storage at −80 °C.

Library preparation and sequencing of both the rRNA gene amplicon samples and the shotgun metagenomes was conducted by the Department of Energy Joint Genome Institute (JGI) as part of the Community Science Program.

Targeted Illumina rRNA gene amplicon libraries were prepared using DOE-JGI iTag Sample Preparation for Illumina Sequencing to access prokaryotic and fungal community profiles. The bacterial 16S V4 region was amplified from total DNA using 515FB (5′-GTGYCAGCMGCCGCGGTAA-3′) and 806RB (5′-GGACTACNVGGGTWTCTAAT-3′) primers^17^ with chloroplast and mitochondrial PNA blocking oligos for 16S endophyte samples (PNA Bio Catalog #MP01-25 and #PP01-25). The fungal ITS2 region was amplified using ITS9_Fwd (5′-GAACGCAGCRAAIIGYGA-3′) and ITS4_Rev (5′-TCCTCCGCTTATTGATATGC-3′) primers^18^. Both forward and reverse primers contained Illumina dual index sequencing adaptors and one 12 bp index. Forward primers contained a spacer sequence of five 5′ degenerate nucleotides (N), and reverse primers contained zero to three 5′ frameshifting nucleotides that provide sequence diversity at the start of sequencing read 1^19^. PCR assays were performed with the 5PRIME HotMaster Mix (Quanta BioSciences, Inc., MD, USA). Sequencing of the flowcell was performed on the Illumina MiSeq sequencer using MiSeq Reagent kits and following a 2 × 300 nt indexed run protocol. We note that the samples BM_ITS2_LN_R12 and BM_ITS2_SN_R11 failed to yield amplicons and, thus, are absent from our data.

Genomic DNA of root and substrate (bulk soil or exposed rocks) microbial communities from three individuals (samples numbered from R1 to R3) of each plant species was used to generate the shotgun metagenome sequencing data. A total of 10 ng of DNA was sheared to 300 bp using a Covaris LE220 (Covaris, MA, USA) and size selected using SPRI beads (Beckman Coulter, CA, USA). The fragments were treated with end-repair, A-tailing, and ligation of Illumina compatible adapters (Integrated DNA Technologies, Inc., IA, USA) using a KAPA-Illumina library creation kit (Kapa Biosystems, MA, USA), and a 5 cycle PCR was used to enrich for the final library. The libraries were prepared for sequencing on the Illumina HiSeq sequencing platform utilizing a TruSeq Rapid paired-end cluster kit, v4. Sequencing of the flowcell was performed on the Illumina HiSeq 2500 sequencer using HiSeq TruSeq SBS sequencing kits, following a 2 × 150 nt indexed run protocol.

### Amplicon sequence variant inference

Raw amplicon sequencing data from *V*. *epidendroides* and *B*. *macrantha* associated communities were retrieved from the DOE-JGI Genome Portal^9^. The paired-end FASTQ files were then deinterleaved using fqtools (version 2.0)^20^ to generate pairs of R1 and R2 FASTQ files which were then inspected using FastQC (version 0.11.7)^21^. Next, the primer sequences were trimmed out of the reads using cutadapt (version 1.16)^22^ keeping only the read pairs that contained the complete sequences of both the forward primer in the R1 read and the reverse primer in the R2 read. Primer sequences with insertions, deletions or error rates greater than 20% were removed. A second quality check was performed with FastQC to obtain Phred score distributions which were used to determine the trimming length that was used in the subsequent variant inference step. FastQC and cutadapt results were summarized in an HTML report with MultiQC (version 1.6)^23^.

Amplicon sequence variants (ASVs) of the 16S and ITS libraries were obtained separately using DADA2’s denoising algorithm (version 1.6.0)^24^. First, the R1 and R2 reads of the 16S samples were truncated to 245 bp and 180 bp, respectively. Reads from ITS samples were not truncated to a fixed length, because this region has significant length variation across genomes. Subsequently, reads were filtered to remove the reads with more than two expected errors and ambiguous bases. Parameters of the error models were obtained by alternating sample inference with parameter estimation until convergence was achieved. The error models and dereplicated reads pooled from all samples were used as input for the dada function to obtain denoised sequences from R1 and R2 reads. Pairs of R1 and R2 reads with a minimum overlap length of 16 bp and no mismatches were then merged to obtain ASVs. Next, PCR chimeras identified with the consensus method were filtered out. Finally, 16S AVSs shorter than 246 bp and longer than 260 bp and ITS ASVs shorter than 50 bp were removed.

### Taxonomic assignment, prevalence filtering and community analysis

Taxonomic assignment of the 16S and ITS ASVs was performed with the DADA2 implementation of the naive Bayesian classifier method^25^. The 16S training dataset consisted of taxonomically assigned sequences from the SILVA database release 132^26,27^, while the ITS training dataset comprised the general FASTA release of the UNITE database version 7.2^28,29^. Minimum bootstrap confidence was set to 50. Exact matching of 16S ASVs to database sequences was used to assign species to these fragments. 16S ASV sequences that were assigned to mitochondria or chloroplast taxa were filtered out.

To remove spurious ASVs, prevalence filtering^30^ was performed using the phyloseq (version 1.22.3)^31^ package. Prevalence was defined as the number of samples in which a given ASV’s abundance was at least 0.01% of the sample read count. ASVs with a prevalence lower than 5% of the number of samples were discarded. The number of reads kept in each sample throughout the steps of sample inference, taxonomic assignment and prevalence filtering can be found in Supplementary Tables S3 and S4 for 16S and ITS, respectively. Finally, the vegan package (version 2.5–3)^32^ was used to calculate Shannon’s entropy^33^ for samples (Fig. 3), which was then divided by the log of the number of ASVs to obtain Shannon’s equitability index.

### Metagenome assembly and annotation

Each of the 12 shotgun sequencing libraries was assembled independently using SPAdes software (version 3.11.1)^34^ in the metagenome mode (--meta), using multiple k-mer sizes (-k 33, 55, 77, 99, 127). Next, the assemblies were processed to remove scaffolds shorter than 150 bp, replace ambiguous nucleotides by N’s, trim trailing N’s and filter out low-complexity sequences using DUST^35^. Contamination from phage PhiX sequences was identified and removed by comparing metagenomic sequences to the PhiX genome using BLASTn^36^. Structural and functional annotation of microbial metagenomes was then performed using the DOE-JGI Microbial Genome Annotation Pipeline (MGAP v.4)^37^, as described below.

Briefly, structural annotation started with the detection of CRISPR sequences using CRT^38^ and PILER-CR (version 1.06)^39^. Transfer RNAs were predicted with the tRNAscan-SE tool (version 1.3.1)^40^ and ribosomal RNAs were predicted using the hmmsearch tool from the HMMER package (version 3.1b2)^41^ to compare metagenomic sequences to a set of internal hidden Markov models (HMMs) generated from an alignment of rRNA genes from several IMG/M bacterial genomes. Other types of noncoding RNAs were detected by comparing the metagenomic sequences to the Rfam 10.1 database^42^ using BLASTn and, subsequently, using cmsearch from the INFERNAL package (version 1.0.2)^43^. Prediction of protein-coding genes was achieved using Prodigal software (version 2.6.2)^44^.

To functionally annotate the metagenomes, protein-coding genes were compared with a diverse set of publicly available functional databases. To assign predicted sequences to Clusters of Orthologous Groups of proteins (COGs), protein sequences were compared with the 2014 release of the COG position-specific scoring matrices (PSSMs) from the CDD database^45^ using RPS-BLAST. Protein-coding genes were also compared with the KEGG gene database (release 71.0) using UBLAST^46^, and the top hits were used to assign KEGG Orthology (KO) terms^47^. KO assignments were then used to designate Enzyme Commission (EC) numbers and, consequently, MetaCyc^48^ reactions to coding genes. Protein family annotations were obtained by searching protein sequences against the Pfam (release 28.0)^49^ and TIGRfam (release 14.0)^50^ databases using the hmmscan tool from the HMMER package. InterProScan (release 48)^51^ was employed to assign additional protein family annotations, namely, SMART, PrositeProfiles, PrositePatterns and SuperFamily. IMG terms^52^ are assigned to genes that have at least two out of the top five hits of a UBLAST search of the IMG database with an IMG term. Finally, signal peptide prediction was performed using SignalP (version 4.1)^53^ software.

Metabolic distinctions between soil and rock and between *V*. *epidendroides* and *B*. *macrantha* microbial communities were appraised by testing for differences in the number of genes associated with each MetaCyc pathway. For this purpose, we used DESeq2 (version 1.20.0)^54^ to normalize data with respect to library size, shrink effect sizes (log2 fold changes), estimate and shrink dispersions and perform a Wald test for each pathway. False discovery rate values were obtained by applying the Benjamini-Hochberg procedure to the p-values provided by the Wald test.

Raw data of shotgun sequencing were deposited in the SRA database^55–66^. Assembled annotated metagenomes were deposited in the DOE-JGI’s Integrated Microbial Genomes & Microbiomes (IMG/M) system^67^ (Supplementary Table S5).

## Data Records

Raw data of both the 16S and ITS amplicon sequencing^9^ and the shotgun sequencing^55–66^ were deposited in the NCBI Sequence Read Archive. Amplicon sequencing data is also available through the Genome Portal (https://genome.jgi.doe.gov/portal/) via the accession IDs provided in Supplementary Table S5. Sample description, BioProject, SRA Study, SRA Run and JGI accessions of each of the sequencing libraries generated in this study are available in Supplementary Table 5. Code used to process amplicon sequencing data was uploaded to the Open Science Framework^68^.

## Technical Validation

The quality and purity of the extracted DNA was assessed using DOE-JGI Genomic DNA Sample QC, which consists of the quantification of nucleic acid concentration using Qubit Fluorometric Quantitation (Thermo Fisher Scientific Inc., MA, USA) and a NanoDrop spectrophotometer (Thermo Fisher Scientific Inc., MA, USA), inspection of the 260/280 and 260/230 wavelength (nm) ratios and analysis by electrophoresis agarose gel. PCR of the 16S and ITS regions was controlled by reviewing the amplicon size and ensuring the absence of contaminations on an electrophoresis agarose gel. The prepared libraries were quantified using Kapa Biosystem’s next-generation sequencing library qPCR kit and run on a Roche LightCycler 480 real-time PCR instrument (Roche, Basel, Switzerland).

## Supplementary Information

### ISA-Tab metadata file

Download metadata file

### Supplementary information

Supplementary Table S1

Supplementary Table S2

Supplementary Table S3

Supplementary Table S4

Supplementary Table S5

Supplementary Figures

## Data Availability

All software used in the computational analysis described above was obtained from the Bioconda project^69^ using the Conda package manager (https://conda.io) and the pipelines were executed through the Snakemake workflow engine^70^. Conda environment files, Snakemake pipeline files and the outputs of each analysis can be accessed through Open Science Framework^68^.
