# Supplementary material for: Microbiomes of Velloziaceae from phosphorus-impoverished soils of the campos rupestres, a biodiversity hotspot
Source: Sci Data. 2019 Jul 31;6:140. doi: 10.1038/s41597-019-0141-3 (PMC6668480; doi:10.1038/s41597-019-0141-3)
Supplement: Supplementary file 7 — Supplementary Figures [file 41597_2019_141_MOESM7_ESM.pdf]

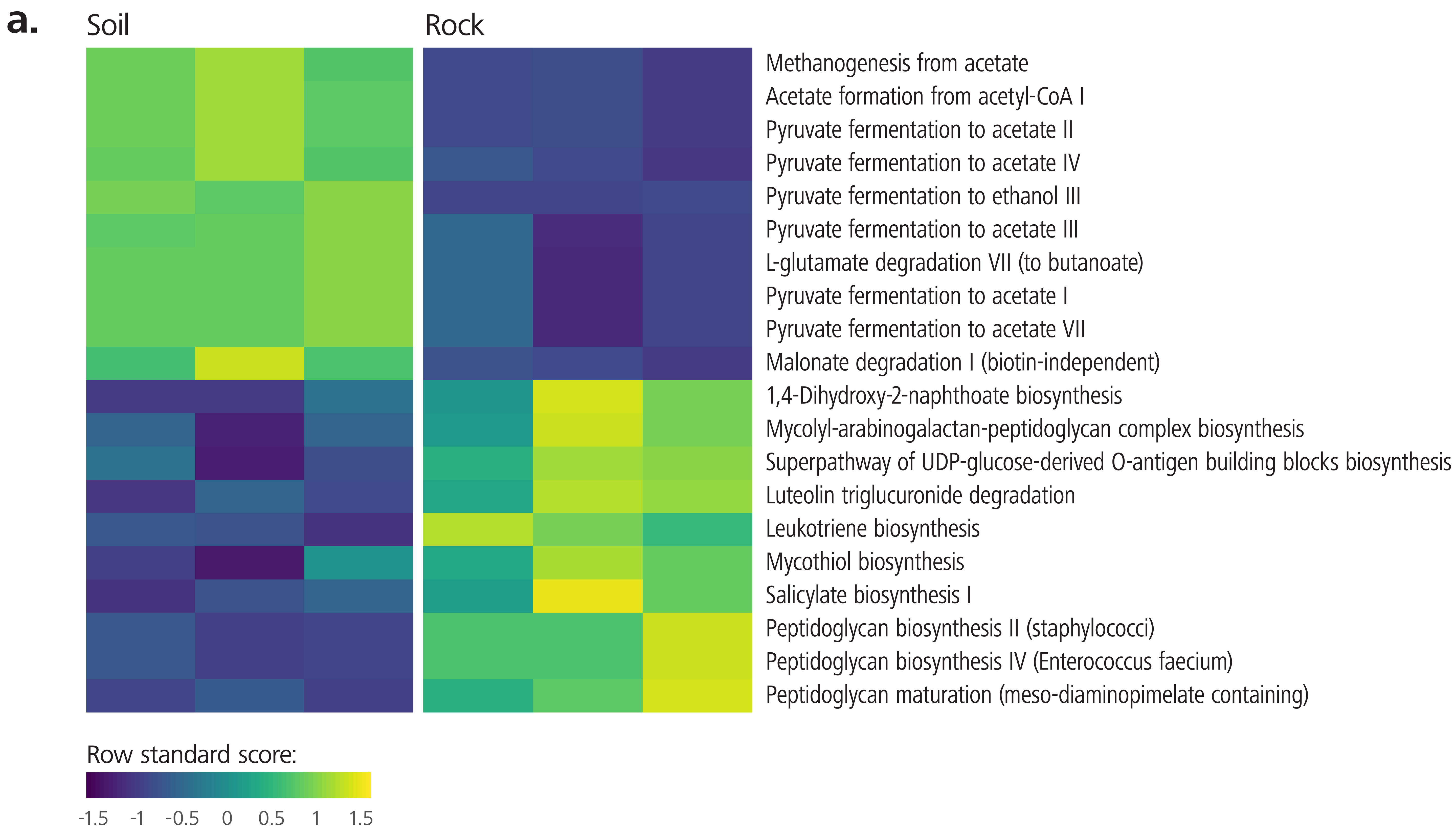

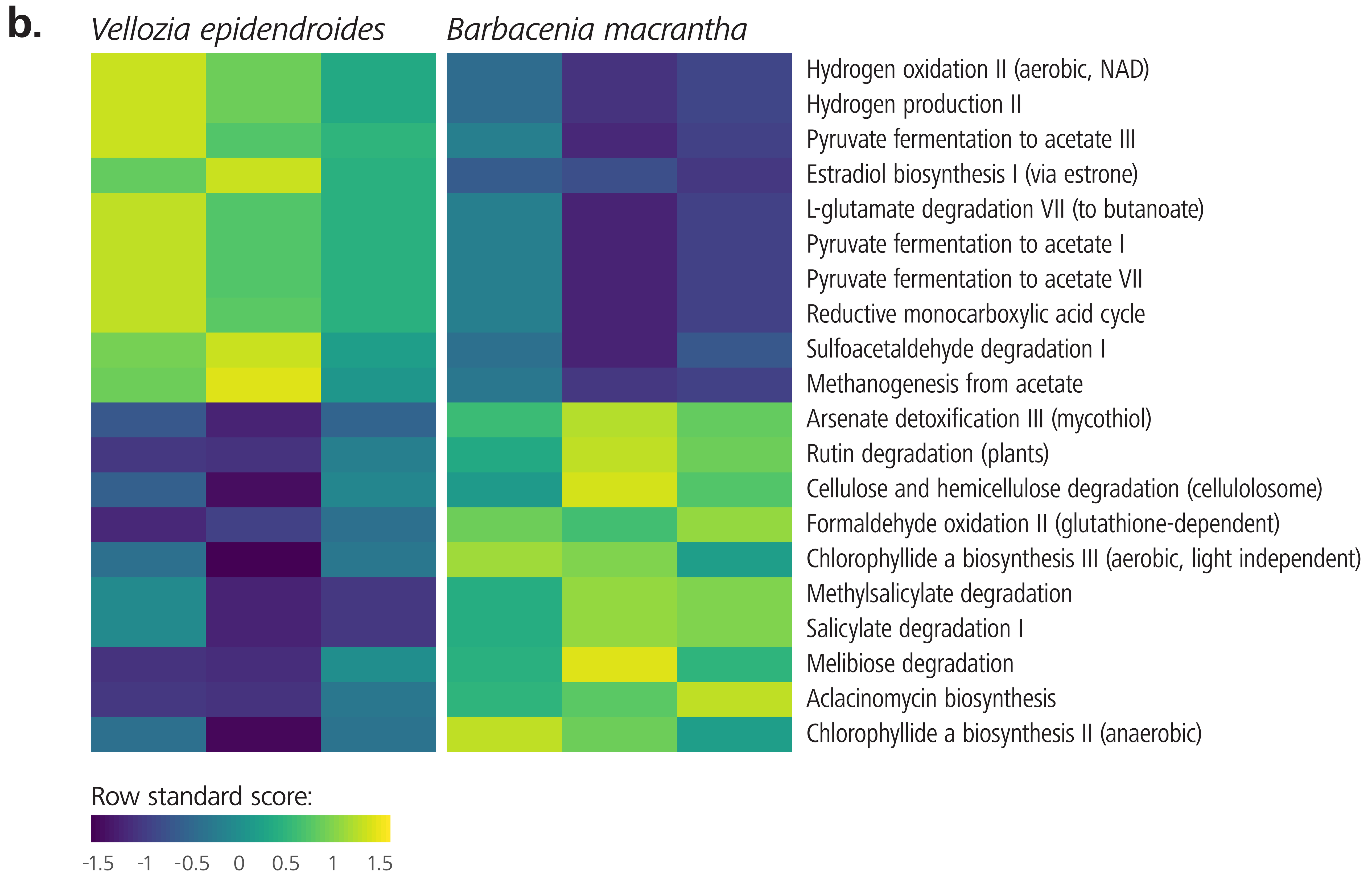

Supplementary Figure S1. The metagenomes of microbial communities found in soil and rock exhibit major metabolic differences. Wald tests were performed to find differentially abundant MetaCyc pathways (a) between the soil and rock metagenomes and (b) between the *V. epidendroides* and *B. macrantha* root metagenomes. For each comparison, effect sizes (log2 fold change) were used for ordering and ten pathways from each end of the rank were selected for represention in heatmaps (a, b). For visualization, count values were transformed using the rlog function and standardized.

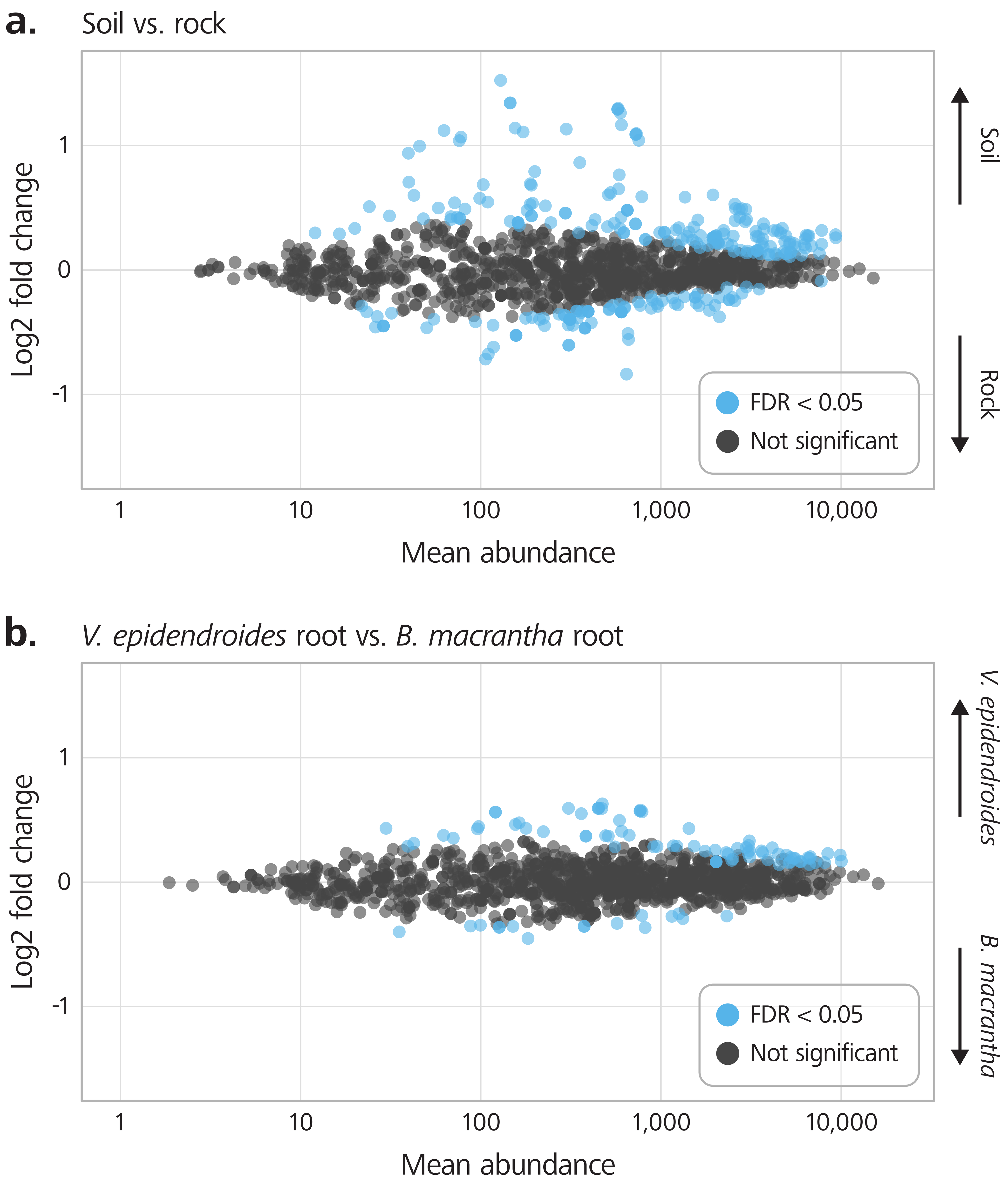

Supplementary Figure S2. Several metabolic pathways are differentially abundant between the studied environments. MA plots showing that a significant fraction of the MetaCyc pathways are differentially abundant (a) between the soil and rock metagenomes and (b) between the *V. epidendroides* and *B. macrantha* root metagenomes. Log2 fold change values were shrunken using a normal prior distribution centered on zero. FDR (false discovery rate) values were obtained by applying the Benjamini-Hochberg procedure to the p-values provided by the Wald test.
